# Mesoscale Dynamics and Niche Segregation of Two *Dinophysis* Species in Galician-Portuguese Coastal Waters

**DOI:** 10.3390/toxins11010037

**Published:** 2019-01-14

**Authors:** Patricio A. Díaz, Beatriz Reguera, Teresa Moita, Isabel Bravo, Manuel Ruiz-Villarreal, Santiago Fraga

**Affiliations:** 1 Instituto Español de Oceanografía (IEO), Centro Oceanográfico de Vigo, Subida a Radio Faro 50, 36390 Vigo, Spain; beatriz.reguera@ieo.es (B.R.); isabel.bravo@ieo.es (I.B.); santi.fraga.ieo.vigo@gmail.com (S.F.); 2 Centro i~mar & CeBiB, Universidad de Los Lagos, 557 Puerto Montt, Chile; 3 Instituto Português do Mar e da Atmosfera (IPMA), Av. Brasília, 1449-006 Lisboa, Portugal; tmoitagarnel@gmail.com; 4 CCMAR, Universidade do Algarve, Campus de Gambelas, 8005-339 Faro, Portugal; 5 Instituto Español de Oceanografía (IEO), Centro Oceanográfico de A Coruña, Muelle das Ánimas s/n, 15001 A Coruña, Spain; manuel.ruiz@ieo.es

**Keywords:** *Dinophysis acuta*, *Dinophysis acuminata*, DSP, physical–biological interactions, niche partitioning, climatic anomaly

## Abstract

Blooms of *Dinophysis acuminata* occur every year in Galicia (northwest Spain), between spring and autumn. These blooms contaminate shellfish with lipophilic toxins and cause lengthy harvesting bans. They are often followed by short-lived blooms of *Dinophysis acuta*, associated with northward longshore transport, at the end of the upwelling season. During the summers of 1989 and 1990, dense blooms of *D. acuta* developed in situ, initially co-occurring with *D.* *acuminata* and later with the paralytic shellfish toxin-producer *Gymnodinium* *catenatum*. Unexplored data from three cruises carried out before, during, and following autumn blooms (13–14, 27–28 September and 11–12 October) in 1990 showed *D. acuta* distribution in shelf waters within the 50 m and 130 m isobaths, delimited by the upwelling front. A joint review of monitoring data from Galicia and Portugal provided a mesoscale view of anomalies in SST and other hydroclimatic factors associated with a northward displacement of the center of gravity of *D. acuta* populations. At the microscale, re-examination of the vertical segregation of cell maxima in the light of current knowledge, improved our understanding of niche differentiation between the two species of *Dinophysis*. Results here improve local transport models and forecast of *Dinophysis* events, the main cause of shellfish harvesting bans in the most important mussel production area in Europe.

## 1. Introduction

Potentially toxic dinoflagellate species of the genus *Dinophysis* are distributed worldwide. To date, around twelve species of *Dinophysis* have been found to produce two kinds of lipophilic toxins: diarrhetic shellfish poisoning (DSP) toxins and/or pectenotoxins (PTXs) [1]. These toxins are retained by filter feeding bivalves and are the main cause of shellfish harvesting bans in western Europe [2]. These bans are enforced when shellfish contamination with DSP toxins and PTXs exceeds the Regulatory Levels (RL) established by European Union directives [3] (herein referred to as “DSP event”). DSP events may occur with moderate cell densities, i.e., a few hundred cells per liter, and blooms of *Dinophysis* (densities > 10^3^ cell L^−1^) are defined as “low biomass blooms of toxin producing microalgae which are transferred through the food web” [1].

**Figure 1:**
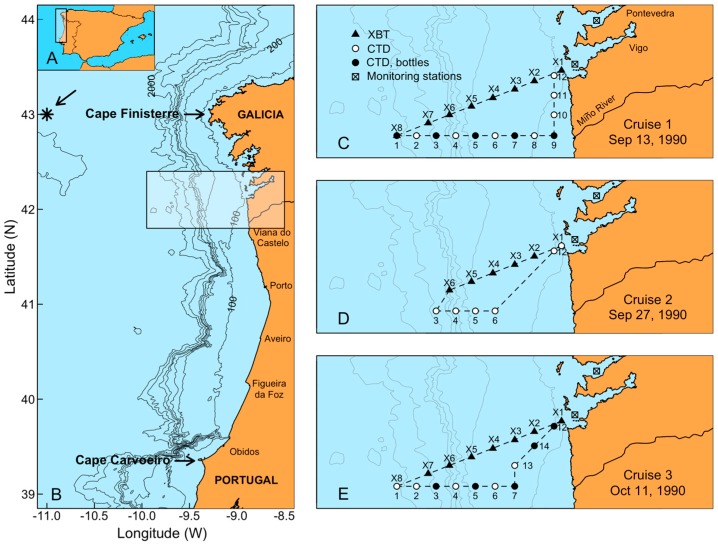
Map of the study area showing (**A**) Iberian Peninsula, (**B**) Northwest Iberia and location of the sampling station (asterisk) for upwelling index estimates, and (**C**–**E**) location of the sampling stations during the three cruises and of the two monitoring stations in Ría de Vigo and Ría de Pontevedra.

Negative impacts of *Dinophysis* blooms, namely of *D. acuminata* and *D. acuta*, are particularly severe in the Galician Rías Baixas and northern Portugal, northwestern Iberia, where harvesting bans may last more than nine months in the most affected shellfish production areas [1,4,5]. This region, located on the northern limit of the Canary Current upwelling system (Figure 1A,B), is subject to a seasonal upwelling regime due to latitudinal shifts of the Azores high- and the Iceland low-pressure systems [6]. Predominant northerly winds from April to September provoke upwelling, and southerly winds from October to March lead to downwelling. In early spring and summer, northerly winds create jets of cold upwelled water on the shelf, and a southward flow of offshore surface waters, the Portuguese Coastal Current (PCC) [7,8] (Figure 1). A poleward countercurrent, the Portuguese Coastal Undercurrent (PCUC), also known as the Poleward Surface Slope Current, or the Iberian Poleward Current (IPC), transports warmer and saltier subtropical water to the north [9,10]. In addition, during the autumn transition from the upwelling to downwelling season, a relatively narrow poleward warm flow has been described on the inner shelf, the “inner shelf countercurrent”, inshore of a southward moving tongue of previously upwelled water [11,12].

Upwelling has been identified as the main physical factor controlling phytoplankton dominance in the Galician Rías [13,14,15] and changes in upwelling patterns related to changes in phytoplankton community composition and in the frequency of toxic algae events [13,14,16]. On a seasonal scale, initiation or intensification of PSP (*Gymnodinium catenatum*) and DSP (*Dinophysis*) events have been associated with upwelling relaxation at the end of the upwelling season. The inner shelf countercurrent has been related with a northward transport of harmful dinoflagellates from northern Portuguese waters towards the Galician Rías Baixas [11,17]. On a smaller spatiotemporal scale, the highest risk of toxic events occurs during relaxation/downwelling between upwelling pulses (transport), or with calm weather and a stratified water column following upwelling (in situ growth) [18,19,20].

Previous studies in the region have shown that *D. acuminata* and *D. acuta* exhibit marked differences in their phenology [21,22,23,24] and occur associated with different microplankton assemblages throughout the annual succession [25]. Thus, the initiation of the *D. acuminata* growth season has been shown to be tightly coupled to the beginning of the upwelling season (March to September) and establishment of a shallow early spring pycnocline [26]. Earlier (March) DSP events caused by this species have been related to anomalous wind patterns the preceding winter [27]. In contrast, *D. acuta*, a mid-to-late summer species in northern Portugal, thrives under thermal stratification combined with moderate upwelling (Figure 1B) [28,29]. High densities of *D. acuta* in the Rías Baixas are usually found only at the end of the upwelling season (autumn transition) associated with upwelling relaxation and longshore transport [21,30,31]. But during exceptionally hot and dry summers combined with moderate upwelling pulses, *D. acuta* was found to grow in the Rías Baixas at the same time as and later replacing *D. acuminata* [23]. In addition, toxic blooms of the chain former *Gymnodinium catenatum*, producer of paralytic shellfish poisoning (PSP) toxins, occurs in some years in the autumn [11,32]. Blooms of *G. catenatum* have been also related to longshore transport at the end of the upwelling season, but a time lag of approximately seven days (two consecutive samplings) was usually observed in the Galician HAB monitoring between the sudden peaks of this species from 1986 to 1990 and the preceding maxima of *D. acuta* (unpubl. data). This time lag suggests different locations of the source populations for each species’ blooms.

In 1990, exceptional summer blooms of *D. acuta*, in terms of cell density and time of co-occurrence with *D. acuminata*, developed in situ in the Galician Rías Baixas [18,33]. Later, during the autumn transition, there were simultaneous blooms of *D. acuta* and *G. catenatum.* Three research cruises were carried out on the Galician shelf to measure physical properties of the sea surface and water column, nutrients and HAB species distribution before, during and after the intense autumn blooms, in addition to the routine monitoring in shellfish production areas. The objective of these cruises was to identify the origin of the inoculum populations of *G. catenatum* [34] and no information was provided about the accompanying populations of *Dinophysis.* Here, unexplored results from these cruises, in addition to monitoring data from the rías of Pontevedra and Vigo and from the northern Portuguese coast are re-examined in the light of current knowledge with a focus on the co-occurring *Dinophysis* species. Results obtained here contribute to parameterize mesoscale environmental conditions associated with exceptional blooms of *D. acuta* developed that year and most important, the niche partitioning between *D. acuta* and *D. acuminata* explaining their spatiotemporal segregation. This information is used to refine local transport models and improve capabilities to forecast toxic events in the Galician Rías Baixas.

## 2. Results

### 2.1. Meteorological and Hydrographic Conditions

**Figure 2:**
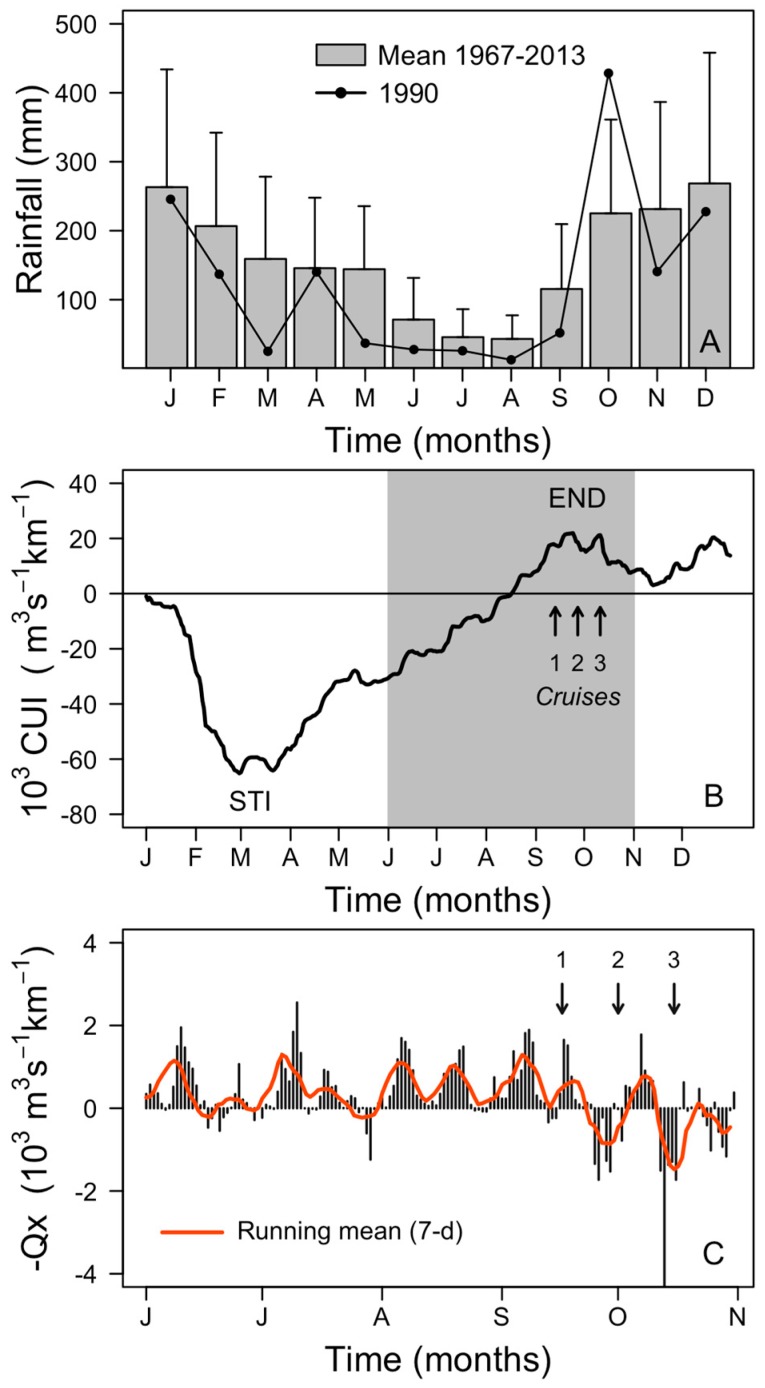
(**A**) Monthly rainfall (mm) in 1990 and the 30-y (1961–1990) monthly mean at Vigo airport. Whiskers indicate standard deviation. (**B**) Cumulative upwelling index (CUI) observed at 43° N in the Canary Current upwelling system in 1990. Upwelling and downwelling transitions are indicated. (**C**) Daily Ekman transport (m^3^ s^−1^ km^−1^) estimated at 43° N, from June to October 1990. Arrows indicate the initiation day of the three shelf water cruises.

Summer 1990 in northwest Spain was extremely hot and dry. Positive air temperature anomalies were +2.6 °C (maximum of 36.6 °C on 20 July) in July and +2.0 °C in August compared with the 47-y (1967–2013) mean. Total rainfall from June to September in 1990 (118 mm) was less than half the mean value (263 mm) for the same period in the last 47-y (Figure 2A). In contrast, a significant positive anomaly was observed in autumn rainfall, with more than double the monthly mean (210 mm) during October (428 mm) (Figure 2A).

Estimates of the Cumulative Upwelling Index (CUI) showed that in 1990, the start of the upwelling season or “spring transition”, on 21 March, was within the normal time-window observed in the climatological mean (1967–2013), but the autumn transition, on 24 September, was two weeks earlier (Figure 2B). Thus, the second cruise was three days after the end of the upwelling season. The Total Upwelling Magnitude Index (TUMI), 81,280 m^3^ s^−1^ km^−1^, from 12 March to 30 September, was slightly above the 47-y mean (69,650 m^3^ s^−1^ km^−1^). At the event scale (7–10 days), the year 1990 was a “normal” year, presenting average patterns in its sequence of upwelling-relaxation cycles during spring and summer (Figure 2C).

**Figure 3:**
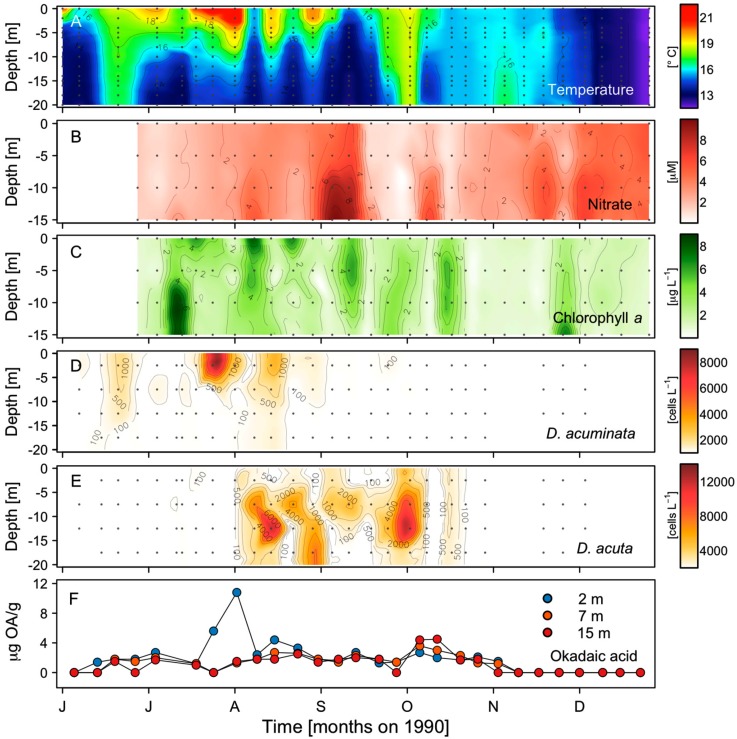
Time series of (**A**) temperature (°C), (**B**) nitrate (μM), (**C**) chlorophyll *a* (μg L^−1^), (**D**) *D. acuminata*, and (**E**) *D. acuta* cell densities (cells L^−1^); (**F**) Okadaic acid in mussels (3 depths) digestive glands (µg g^−1^ HP), from June to December 1990 at a monitoring station (P2) in Ría de Pontevedra. Isotherms are drawn at intervals of 1 °C. Gray dots indicate depth and time of measurements. *Dinophysis* contour plots were made with cell density estimates from integrated (0–5 m, 5–10 m, and 10–15 m) tube samples plotted at 2.5, 7.5, and 12.5 m.

From mid-June to early August the top 10 m of the water column were thermally stratified (Figure 3A). Stratification and sea surface temperature (SST) (22 °C) reached record values for the area in late July [18,33]. During August, there was evidence of a strong (1700–2200 m^3^ s^−1^ km^−1^) upwelling pulse (SST 15 °C, 10 µM nitrates at 15 m) after the first week followed by intermittent intrusions of colder water and increments of nitrate levels alternated with periods of rewarming and increased stratification that were not as marked as in July (Figure 3B). These intermittent upwelling pulses were followed by significant increases of chl *a* concentrations with a maximum value of ~8 μg chl *a* L^−1^ at the surface on 8 August (Figure 3C). During the last third of September, nitrate levels declined to almost undetectable levels, bottom temperatures increased, and progressive mixing took place in response to a few days of upwelling relaxation before downwelling. These conditions are common in the area at the end of the upwelling season, which in 1990 occurred two weeks earlier than the 47-y mean.

### 2.2. Seasonal Variability of *Dinophysis* Species and Microphytoplankton in Ría de Pontevedra

During June and the first half of July, moderate (10^2^–10^3^ cells L^−1^) densities of *D. acuminata* were observed in the warmer top 0–5 m layer (Figure 3D). Maximal densities were found on 23 July (8.3 × 10^3^ cell L^−1^) in the same layer, followed by almost undetectable levels after the strong upwelling pulse (2200 m^3^ s^−1^ km^−1^) in early August. A second surface maximum developed by mid-August (2.2 × 10^3^ cell L^−1^). A new decline followed, very low numbers were detected in September, and cells of *D. acuminata* were no longer detected either in the hose or in net samples in October. *Dinophysis acuta*, first detected on 10 July, exhibited low densities (max. 160 cell L^−1^) that month. Rapid growth took place in early August, with a maximum of 14 × 10^3^ cell L^−1^ found at 10–15 m on 13 August (Figure 3E), co-occurring with the second peak of *D. acuminata* at the surface. The depths of *D. acuta* maxima followed the vertical excursions of the isotherms. A second peak of 13.8 × 10^3^ cells L^−1^ occurred at 10–15 m on 2 October following downwelling and the species was no longer detected after 22 October (Figure 3E).

**Figure 4:**
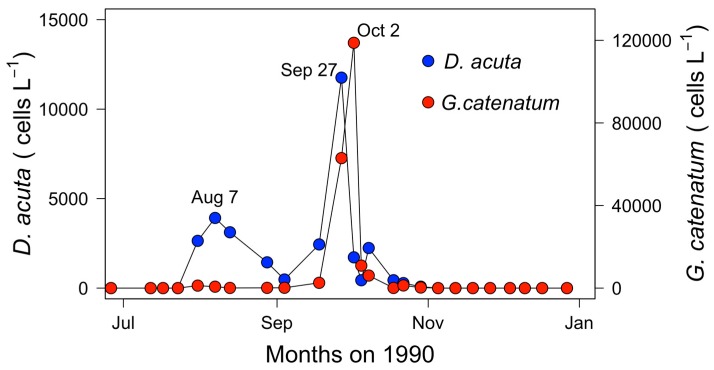
Distribution of *D. acuta* and *G. catenatum* cell maxima in the vertically integrated (0–5, 5–10, and 10–15 m) samples from a monitoring station at the mouth of Ría de Vigo (Figure 1).

During June, small centric colony-forming diatoms (*Leptocylindrus minimus*, *Leptocylindrus danicus*, *Guinardia delicatula*, and *Dactyliosolen fragilissimus*) represented over 90% of the microphytoplankton accompanied by nanoplanktonic flagellates. In early July, *Pseudo-nitzschia seriata*-group species constituted >87%, and *Tripos fusus* was the most abundant dinoflagellate. In the second half of July, during maximal stratification, red patches of the ciliate *Mesodinium* cf *rubrum* were observed on the surface at noon, and *Proboscia alata*, and to a lesser extent *Pseudo-nizschia* spp., *Leptocylindrus* spp., and *T. fusus* were the most abundant species in the samples. Diatoms, in particular *P. alata*, *Rhizosolenia shrubsolei*, *L. danicus*, and *L. minimus*, were still dominant (>95%) at the three depth intervals all through August. The last two diatoms were dominant in the top 10 m in September, while *Gymnodinium* spp. were the most abundant in the 10–15 m layer at the end of that month. Thus, from June to September, when nutrients were high (Figure 3B), diatoms and small flagellates predominated and *Dinophysis* species (*D. acuminata* + *D*. *acuta*) represented a small proportion (1–5%) of the microphytoplankton community. The situation changed abruptly on 2 October, following some days of upwelling relaxation, when a sudden peak of *G. catenatum,* co-occurring with *D. acuta,* became the main component of a dinoflagellate (*T. fusus*, *Protoperidinium divergens*, and *Prorocentrum triestinum*)-dominated microplankton community with no diatoms. There was a lag of approximately five days between the cell maxima of *D. acuta* and *G. catenatum* at the monitoring station in the mouth of Ría de Vigo (Figure 4). After 8 October, diatoms reoccurred and together with small flagellates were the main component of a very sparse phytoplankton population.

### 2.3. Distribution of OA with Depth in Raft Mussels

Results from the monitoring of okadaic acid (OA) at three depths of the mussel ropes showed low levels of OA (1.4 µg g^−1^ HP) in mussels from 7 and 15 m in early June, and a moderate progressive increase (up to 3 µg g^−1^ HP) during June until mid-July. Between 17 and 31 July, OA levels at 2 m increased, coinciding with the surface maximum of *D. acuminata*, to the highest value of the season (10.8 µg g^−1^ HP). In August (1.9–3.0 µg g^−1^ HP) and September (1.5–1.8 µg g^−1^ HP), there was an even distribution of the toxin with depth, with the exception of a small peak (4.4 µg g^−1^ HP) at 2 m coinciding with the second maximum of *D. acuminata* before the population declined. A new increase with a maximum at 15 m (4.7 µg g^−1^ HP) was detected at the same time and depth as the peak of *D. acuta*. From 8 October onward, OA levels gradually decreased, becoming undetectable by the end of the month at 15 m and on 5 November at 2 and 7 m.

Assuming for *Mytilus galloprovincialis* an average whole flesh:digestive gland weight ratio of 11:1 [35] and that approximately 80% of the toxins are accumulated in the digestive gland, the maximum level of OA observed in late July would be equivalent to approximately 1230 µg OA 100 g^−1^ meat, i.e., 7.7-fold higher than the RL.

### 2.4. Hydrodynamic Conditions on Shelf Before, During, and after the Autumn DSP and PSP Events

**Figure 5:**
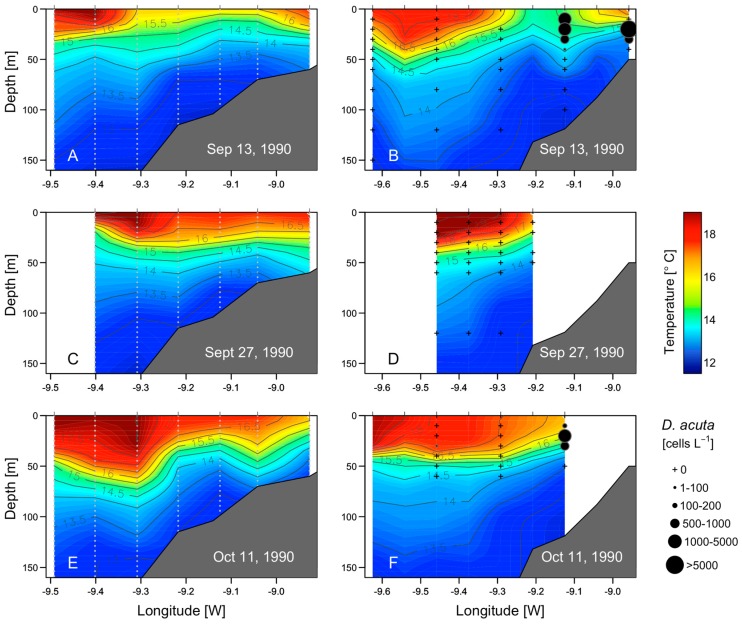
Vertical distribution of (**A**,**C**,**E**) temperature (°C), measured with XBT, in transects diagonal to the coast (left) and (**B**,**D**,**F**) temperature (CTD casts) and *D. acuta* cells density (bottle samples) in transects perpendicular to the coast (right) sampled during the three cruises on the Galician shelf (see Figure 1).

**Figure 6:**
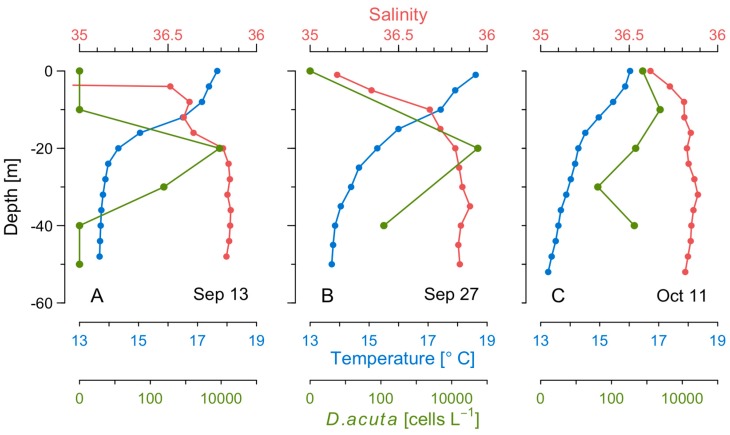
Vertical profiles of temperature (blue), salinity (red), and *D. acuta* cell densities (green), on the adjacent shelf during three cruises on (**A**) 13–14 September, (**B**) 27–28 September, and (**C**) 11–12 October.

Cruise 1 (13–14 September). During the first cruise, there was offshore Ekman transport and upwelling associated with the onset of northerly winds the preceding days (Figure 2). The cruise coincided with an intense intrusion of cold water in bottom layers into the rías and with the export of surface ría waters to the shelf (Figure 5A). The upwelling pulse in the shelf–rías system was characterized by a marked upwelling front about 19 nm off the coast (~150 m isobath) with a gradient of 2.3 °C in 1.6 nm (Figure 5 A,B). The phytoplankton community on the inshore side of the front was dominated by the same diatoms than inside the rías, i.e., *L. danicus*, *L. minimus*, and by species of *Pseudo-nizschia seriata*-group spp. Seaward of the front there was a sharp decline in chlorophyll *a* fluorescence, and a dominance of small flagellates; values of salinity (>35.9) and temperature (>18 °C) corresponded to those typical of the Iberian Poleward Current (IPC) [9]. *D. acuta* cells were only observed at stations on the inshore side of the upwelling front. Maximum cell densities (9 × 10^3^ cell L^−1^) were observed at the base of the pycnocline (20 m) on a shelf station (50 m isobaths) close to the mouth of the Miño River (station 9) (Figure 5B and Figure 6A).

Cruise 2 (27–28 September). Following some days of downwelling relaxation, a low pressure system off the western Iberian peninsula caused a shift from northerly to southerly winds on 25 September (Figure 2) resulting in onshore transport of the warmer (19 °C), more saline water located seaward of the front in the previous cruise, and lowering of the pycnocline over the whole shelf to the outer reaches of Ría de Vigo (Figure 5C). Maximal densities of *D. acuta* (5.4 × 10^4^ cells L^−1^) associated with marked vertical gradients (3.3 °C/20 m) were found at the base of the pycnocline at 20 m on a shelf station (station 12, 100 m isobath) close to the southern mouth of Ría de Vigo (Figure 6B). *G. catenatum*, below detection levels in the previous cruise, reached a maximum of 6.2 × 10^4^ cells L^−1^ at 10 m at the monitoring station in the mouth of Ría de Vigo.

Cruise 3 (11–12 October) Renewed northerly winds at the beginning of October, after an intense upwelling event, led to positive Ekman transport (maximum value, 2000 m^3^ s^−1^ km^-1^ on 7 October) and inflow of cold nutrient rich waters into the rías from below with surface outflow of warmer, less saline water from the rías. This re-established a strong thermal stratification and coastward shoaling of the 13.5–16.5 °C isotherms that reached the surface at the mouth of Ría de Vigo (Figure 5E). A new upwelling front developed, much closer to the coast than the one observed during the first cruise (Figure 5E,F). These conditions coincided with the decline of *G. catenatum.* Maximum values of 1–5 × 10^2^ cells L^−1^ of this species were detected in the mouth of Ría de Vigo and adjacent shelf stations. Bloom levels (>10^3^ cells L^−1^) of *D. acuta* persisted at all stations sampled, with cell maxima at 10 m, below the warmer and saltier surface layer (Figure 6C).

### 2.5. Thermohaline Conditions Associated with *Dinophysis* and *G. catenatum* Shelf Maxima

**Figure 7:**
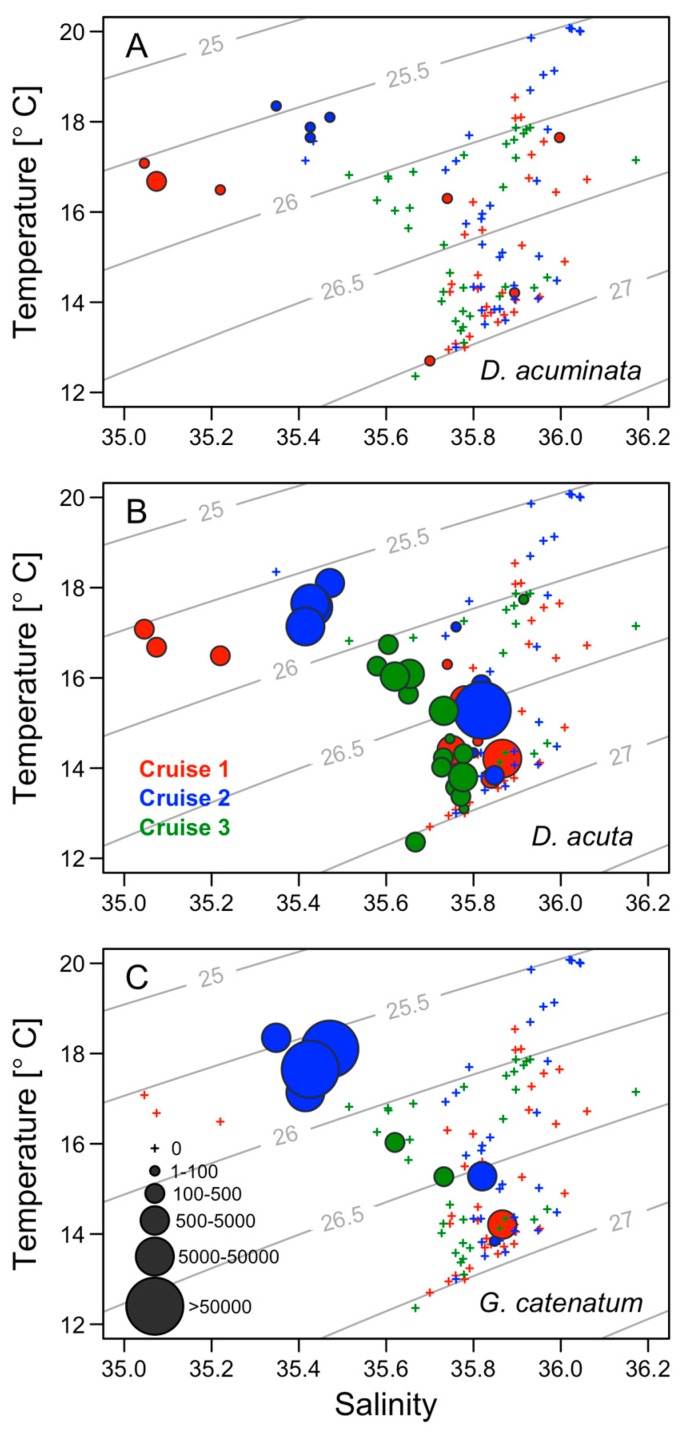
Cell densities (cells L^−1^) of (**A**) *D. acuminata*, (**B**) *D. acuta*, and *(***C**) *G. catenatum* plotted over TS diagrams from the three shelf cruises from September to October 1990. Contour lines (gray) represent isopycnals spaced at intervals of 0.5 σ.

Cell densities of toxigenic species *(D. acuminata*, *D. acuta*, and *G. catenatum*) plotted over TS diagrams during the three cruises showed that cell maxima of the three species were located in the mixed surface layer (< 30 m). This water layer is delimited by a seasonal thermocline (Figure 7). *D. acuminata* was detected in low densities (max. 120 cells L^−1^) during the first and second cruises which showed salinities <35.5 and temperatures of 16 to 18 °C (Figure 7A). Plots of *D. acuta* cell densities on TS diagrams showed that it was most abundant in a salinity range of 35.4 to 35.9 and a temperature of 14 to 18 °C (Figure 7B). The cell maximum (5.4 × 10^4^ cells L^−1^) observed during the second cruise was associated with the 26.5 *σt* isopycnal (Figure 7B). In the case of *G. catenatum*, highest cell densities were associated with a temperature of 14 to 18 °C and salinity 35.4 (Figure 7C). This water mass, although similar in temperature to the warm offshore water, had a lower salinity.

**Figure 8:**
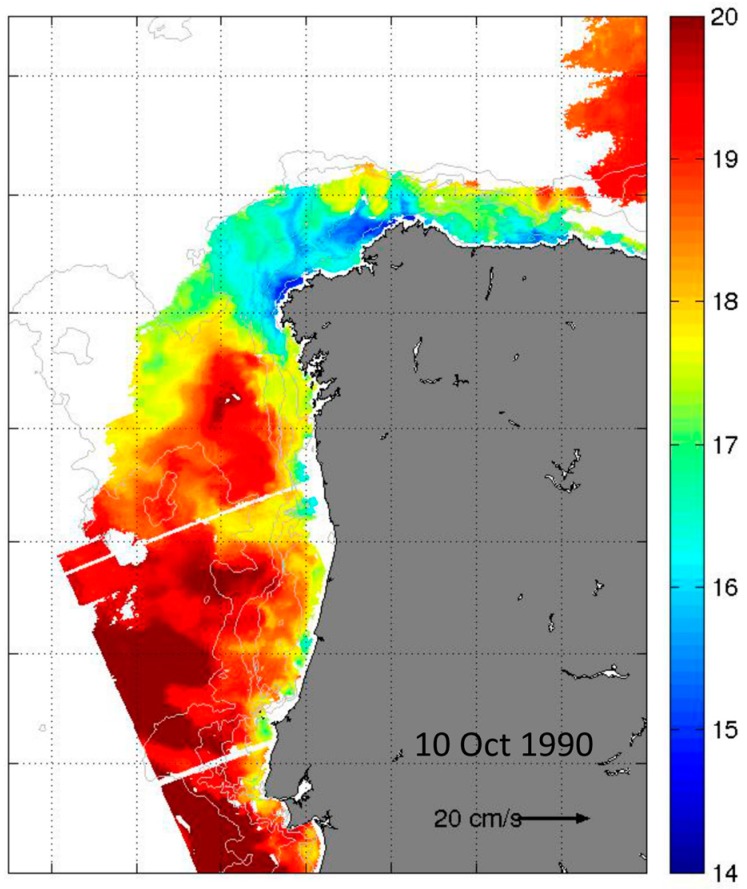
Sea Surface Temperature (SST) from AVHRR (2-km) satellite data on 10 October 1990. White patches represent clouds.

Images from the AVHRR sensors, corresponding to the day pass of the satellite over the study area on 10 October revealed a surface poleward flow characterized by SST values >18 °C (Figure 8), which correspond to the signature of the Iberian Poleward Current, IPC. These agree with the surface salinity (>35.9) and temperature (>18 °C) values observed at the offshore stations (stations 1 and 2) on 11 October during the third cruise.

### 2.6. Mesoscale Dynamics of *D. acuta* in Galician-Portuguese Coastal Waters

**Figure 9:**
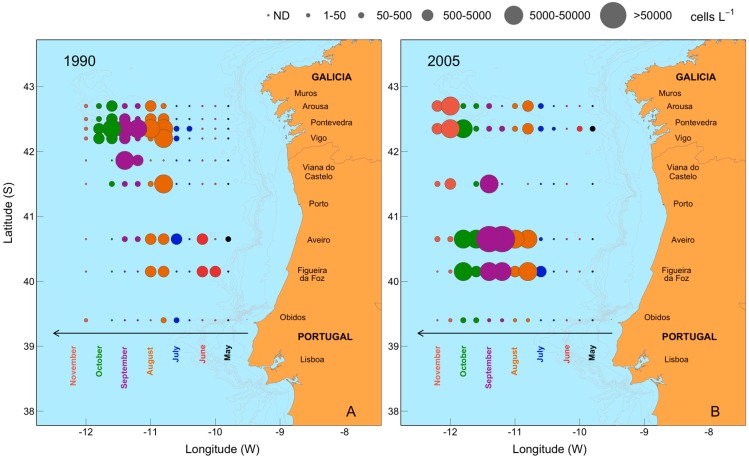
Seasonal variability, from June to November, of *D. acuta* cell maxima at monitoring sites in Galicia and northern Portugal in 1990 (**A**) and 2005 (**B**). Isobaths are shown in gray. The 2005 map is modified from Escalera et al. [30].

Mesoscale dynamics of *D. acuta* cell density distribution estimated from weekly monitoring sampling at different sites along northwestern Iberian coastal waters, from Cape Carvoeiro, Portugal to Cape Finisterre, Spain between July and October 1990, were compared with the distribution in the same area observed in 2005 (Figure 9). In July 1990, low to moderate density (10^2^–10^3^ cells L^−1^) populations of *D. acuta* were detected throughout Galician-northern Portuguese coastal waters. Densities progressively increased reaching a maximum off Aveiro (2.9 × 10^4^ cells L^−1^) on 30 July and off Ría de Vigo (3.5 × 10^4^ cells L^−1^) on 13 August. 

From mid-August onwards, the Galician Rías became the center of gravity (the region of highest population density) of the late summer *D. acuta* population distributed from Óbidos, Portugal to the Galician Rías. This population showed a seasonal bimodal distribution in the Galician Rías, with a second maximum of 2 × 10^4^ cells L^−1^ observed in Ría de Pontevedra in early autumn (24 September) (Figure 9A). At the same time, cell densities 1–2 orders of magnitude lower (<1 × 10^3^ cells L^−1^) were detected off Aveiro. From 8 October onwards, cell densities declined, and they were below detection levels at most stations by the end of the month. Thus, in 1990 the growth season of *D. acuta* started and finished earlier in northern Portugal and showed a bimodal pattern with an unusual summer growth on the Galician coast, where the center of gravity of the population was located throughout August and September. The situation was quite different in 2005, a year which exhibited the most typical seasonal pattern of *D. acuta* populations in northwestern Iberia. In 2005, *D. acuta* populations developed in Portuguese coastal waters in summer, reaching record values off Aveiro in late August, and declined in late October. On the Galician coast, *D. acuta* densities were extremely low in summer. High densities were detected in late October associated with northward longshore transport by the end of the upwelling season [30] (Figure 9B). Therefore, during 2005, the center of gravity of the summer distribution of *D. acuta* population was off Aveiro, which is the common situation for the seasonal distribution of this species [28,29]. 

## 3. Discussion

HAB species respond to changes in local hydrodynamics that may be driven by large-scale atmospheric processes. Nevertheless, knowledge about the time scales over which preceding conditions shape communities and their biomass is scarce [36]. In 1990, the seasonal spatial variability of *D. acuta* on the Galician northern Portuguese shelf showed a northward drift and was characterized by unusually early dense (>10^4^ cells L^−1^) summer blooms. These blooms were associated with exceptional hydroclimatic conditions in summer, including positive anomalies of SST (over 2 °C) on the Galician shelf and in the Rías. These anomalies were a large scale phenomenon which also affected plankton communities in the North Sea and other northeast Atlantic coastal regions [37,38]. A second “normal” bloom occurred at the end of the upwelling season co-occurring with a PSP event of *G. catenatum*. Although both *D. acuta* events (late summer and early autumn) during 1990 reached similar population densities (2–3 × 10^4^ cells L^−1^), they developed under distinct meteorological and oceanographic conditions. The hydroclimatic process implicated in the onset, development, and decline of these exceptional events is discussed here with the overall objective of “identification of key past events which will be re-analyzed and used for training the modelling system”.

### 3.1. Initiation of *D. acuta* Summer Bloom and the Replacement of *D. acuminata*

HABs may be triggered by different mechanisms promoted by physical, chemical and biological conditions optimal for bloom development [39]. In 1990, the exceptional *D. acuta* summer bloom coincided with extreme climate anomalies, characterized by very hot and dry summer conditions. These local weather conditions were accompanied by an upper-level high-pressure anomaly in late July and early August (data not shown). Likewise, Cloern et al. [36] reported extreme climate anomalies associated with an exceptional bloom of the red tide forming dinoflagellate *Akashiwo sanguinea* in San Francisco Bay during summer 2004. Nevertheless, similar summer blooms have not been observed in the Galician Rías since then, despite new records of high summer temperatures.

Recently, Díaz, et al. [33], based on a 29-y time series (1985–2013) analysis of monitoring data from the Galician Rías, suggested that a long period of stable, thermally-driven stratification is necessary for in situ development of summer populations of *D. acuta*. These authors suggested that exceptional in situ development of these populations (mainly July–August) appeared related to an optimal combination of SST (>17 °C), water column stability (>6 weeks) and values of upwelling close to the historic mean. These conditions would keep stability in the stratified top layer down to a favorable depth for *D. acuta*. In summer 1990, as well as in 1989, these “optimal environmental conditions” were observed. Recent laboratory studies have shown that *Dinophysis* species, including *D. acuta*, are obligate mixotrophs which require live ciliate prey (e.g., *Mesodinium* spp.) and light for sustained growth, but they are also able to survive for long periods of time (up to two months) without prey [40,41]. Nevertheless, predator and prey have different environmental requirements, their populations only coincide occasionally [42], and *Dinophysis* populations may often be prey-limited [43]. Monitoring data reported a dominance of *Mesodinium* cf *rubrum* within the microplankton community during the second half of July. Thus, the exceptional summer bloom of *D. acuta* in 1990 may be understood as a local response to an optimal coupling of physical (persistent thermal stratification) and biological conditions (prey availability) promoting in situ growth on the Galician shelf.

The sequential development of *D. acuminata* and *D. acuta* populations, the former with a much longer growth season than the second, is observed in all the geographic areas where these two HAB species commonly occur [44]. This is the case in northwestern Iberia, where a wider continental shelf enhances stratification and the development of dense populations of *D. acuta* in summer with a center of gravity off Aveiro [28]. But in the Galician Rías, in situ growth in summer is very weak (if it is present at all) and DSP outbreaks associated with this species are in the autumn, at the end of the upwelling season and due to longshore transport and accumulation [30]. During the exceptional years (1989 and 1990) described above, *D. acuta* exhibited a seasonal bimodal distribution characterized by two annual peaks. The first maximum, in late summer, associated with in situ growth and the second maximum in the autumn transition linked to physical transport [30].

A detailed understanding of the species-specific processes involved in the replacement of *D. acuminata* by *D. acuta* in late summer during exceptional summer conditions has not so far been achieved. A plausible explanation was given by Escalera et al. [23], who suggested that in the Galician Rías Baixas this replacement appeared to be associated with the establishment of deeper thermoclines. These authors described the 2003 scenario, with a high temperature (~20 °C) in the top layer (1–5 m) during a very hot summer. *Dinophysis acuta* was present and replaced *D. acuminata*, but at very low cell densities. The year 2003 was also characterized by having extremely weak upwelling pulses. This situation was recently reinterpreted in the light of new knowledge on *Dinophysis* feeding-behavior [33]. The low intensity upwelling pulses and subsequent low nutrient levels in the euphotic layer in 2003 would have prevented the development of high densities of *Mesodinium,* and its cryptophyte prey, both part of the food chain required to promote *Dinophysis* growth.

### 3.2. Niche Partitioning and Specific Requirements of D. acuminata and D. acuta

Species can differentiate their niche in many ways, such as by consuming different foods, or using different parts of the environment. The spatial and temporal complexity of upwelling dynamics can create a variety of niche opportunities for phytoplankton populations, including HAB species. In these systems, phytoplankton populations are much more dependent of turbulence (physical control) and nutrient availability [45]. Further, the large species diversity observed indicate that the adaptations and behavioral strategies are varied [46]. Recently, Smayda [47] suggested that different morphological traits allow dinoflagellates exploit the complex niche structure of upwelling systems without the need for special adaptations. 

In this work we studied the population dynamics of two dinoflagellate species of *Dinophysis*—*D. acuminata*, and *D*. *acuta*—which are both kleptoplastic mixotrophs, i.e., they perform photosynthesis with “stolen” plastids from their prey. The two species have identical partial (23 S rDNA) sequences of their plastid *psbA* gene and both are successfully cultivated in the laboratory with the phototrophic ciliate *Mesodinium rubrum* [48]. Therefore, if the two species share the same prey, they would not be able to co-occur unless they occupied different positions in the water column. Results here show that when *D. acuminata* and *D. acuta* coincided in time (August 1990), their maxima occupied different water masses, suggesting a “niche partitioning” with depth. This vertical segregation may be associated with the species-specific response to environmental factors, such as light (quality and intensity) and turbulence.

Recent laboratory experiments with the two species have shown that *Dinophysis acuta* is more susceptible to photodamage, under high light intensities (370–650 μmol photons m^−2^ s^−1^) than *D. acuminata*, but survives better with low light (10 μmol photons m^−2^ s^−1^) and endures longer periods (28 d) in the dark [49]. *D. acuta* is better adapted to low light intensities and photosynthesizes better with blue light, the only wavelength reaching the lower limit of the euphotic zone, and its swimming capacity [46] enables it to succeed in deeper pycnoclines than *D. acuminata.* These features might explain its vertical distribution in summer in the Galician Rías, close to or associated with the pycnocline, whereas *D. acuminata* cell maxima aggregate nearer the surface. The vertical distribution of OA on the raft mussel ropes, with a marked peak at 2 m, provided evidence of the aggregation of *D. acuminata* near the surface in late July. From mid-August onwards, when *D. acuta* became the dominant *Dinophysis* species in the rías, the similar levels of OA in mussels from the three depths sampled suggests this species performed a daily vertical migration.

Morphologically, *D. acuminata* and *D. acuta* are also quite distinct. *D. acuta*, with a biovolume 3 times larger than *D. acuminata,* is much more dorsoventrally compressed than *D. acuminata*, which is rounded. These differences enable *D. acuminata* to endure higher values of turbulence near the surface. In contrast, *D. acuta* moves in layers, close to the pycnocline, with decreased rates of kinetic energy dissipation (ε). Experimental work with cultures of the two species subject to three different levels of turbulence confirmed that *D. acuta* was more sensitive to high levels of turbulence than *D. acuminata* [50]. All these differences in morphology and adaptations to distinct environmental conditions would define the realized niche of each species of *Dinophysis* and justify their co-occurrence in time but at different levels in the water column, even considering their competition for the same prey.

### 3.3. Inoculum Source for Bloom Development

Results from northern Portugal shelf waters have shown that the highest cell densities of *D. acuta* always occurred at the inner-shelf margin. [51]. The best documented example was reported by Moita et al. [29] who described an intense bloom of *D. acuta* (5 × 10^4^ cells L^−1^) restricted to a subsurface thin layer (between 18 and 20 m depth) within the pycnocline extending 30 km offshore.

The three cruises discussed here were originally planned in 1990 to investigate the origin of the inoculum population leading to abrupt increments of *G. catenatum* in the Galician Rías during relaxation at the end of the upwelling season [32]. One hypothesis was that the inoculum for *G. catenatum* blooms was transported by the Iberian Poleward Current. Results here showed that the phytoplankton in the IPC was mainly composed of small flagellates and that *D. acuta* and *G. catenatum* were always found at shelf stations close to the coast, but not at offshore stations. Early suspicions of longshore transport of *G. catenatum* came after observations on the mesoscale dynamics of blooms of this species in 1985 and 1994. Populations of *G. catenatum* were detected in retention areas formed on the lee side of upwelling plumes off Capes Roca and Carvoeiro during summer before blooming in northwestern Iberia in the autumn [52]. These autumn blooms were very sudden, during upwelling relaxation, and were interpreted as a result of advection from shelf populations into the Galician Rías [32]. Twenty years later, improved knowledge on the hydrodynamics and the development of predictive transport models in northwestern Iberia have provided a clearer picture of the mesoscale circulation at the end of the upwelling season and the identification of a poleward inner coastal current [17,53]. This inner poleward current has been associated with the northward transport of *D. acuminata* and *G. catenatum* populations [11], a view supported by observations from Escalera et al. [30] during the intense 2005 bloom of *D. acuta* in the Galician Rías. Estimates of in situ division rates of *D. acuta* throughout its seasonal occurrence that year showed that during the autumn bloom, cells were not dividing at all, so the rapid increase in net growth had to be the result of transport rather than in situ growth. Running of a local hydrodynamic model with data from the autumns of 2005 and 2013 confirmed a northwards advection in an inner shelf current as a plausible mechanism of northwards transport of *D. acuta* from Portugal to Galicia [53]. Surveys in the Celtic Sea, southwestern Ireland, provided evidence of the direct transport of a high-density patch of *D. acuta*, forming a subsurface thin layer within a coastal jet along the south coast of Ireland; the 5-m thick thin layer was centered at 20 m depth and did not coincide with the deeper (30 m) chlorophyll maximum [54]. The main unresolved issue with *Dinophysis* blooms and their contamination of shellfish with DSP toxins in Ireland was the identification of their source. Recent surveys have given evidence for extensive *D. acuta* bloom development in summer in the productive region close to the Celtic Sea Front, a tidal front extending from southeast Ireland to Britain [55]. Therefore, the source population of *D. acuta* would be about 300 km away from the aquaculture sites in Bantry Bay where their impact is maximal, i.e., a similar distance than that from the Aveiro “center of gravity” of *D. acuta* distribution in northwestern Iberia to the intensive mussel aquaculture sites in the Galician Rías Baixas. Likewise, the formation of a tidal front in the warm season has been pointed to as an essential requirement for the development of *D. acuta* blooms in the Firth of Clyde in western Scotland [56]. In the case of the Iberian blooms, the upwelling front at the time the cruises took place established the borders between oceanic water populations dominated by small flagellates, and those in the nutrient-rich shelf waters dominated by microplanktonic dinoflagellates and diatoms. The toxic dinoflagellate populations of concern were distributed on the inner shelf waters, far from the front. A much earlier cruise would have been needed, before bloom initiation, to explore the origin of the inoculum. Nevertheless, results on the distribution of scattered cells of *D. acuta* during the *Morena* cruise in May 1993 confirmed the prebloom distribution of the “pelagic seed banks”, sensu Smayda [57] of this species were restricted to the northern half of the Portuguese shelf [58].

### 3.4. OA Distribution with Depth in Raft Mussels. Implications for Shellfish Exploitation

The sequence of *D. acuminata* (June-early August) and *D. acuta* (August–October) events observed in the Galician Rías in 1990 caused mussel harvesting bans from 9 July to 17 November, with significant economic losses [59]. Based on the vertical distribution of OA and *Dinophysis* species presented here (Figure 4E), it was concluded that *D. acuminata* blooms were associated with mussel toxicity from June to early August, and those of *D. acuta* with the late summer to autumn toxicity. It was also suggested that the smaller-sized *D. acuminata* had a stronger toxic potential than the larger *D. acuta.* We draw special attention to the fact that only OA was measured in the HPLC analyses performed in the 1990s, and that according to the analyses of picked cells from the region [60], it was assumed that OA was the only toxin present in *D. acuta* strains from Galicia. Dinophysistoxin 2 (DTX2) was not described until 1992 [61] and the widespread presence of PTX2 in Galician shellfish during blooms of *D. acuta*, *D. caudata*, and *D. tripos*, until 2002 [62]. *Dinophysis acuta* has a complex toxin profile including OA, DTX2, and PTX2 in addition to small amounts of OA diol-esters and PTX11. It is also known that different years may bring strains with different toxin profiles [63]. In any case, it is certain that the toxin content in mussels exposed to the *D. acuta* bloom in the Galician Rías Baixas in 1990 was much higher than the estimates given at the time. 

A remarkable difference was observed in the vertical distribution of OA during the cell maxima of *D. acuminata* and *D. acuta*. The overwhelmingly higher values of toxin content in surface (2 m) mussels in late July and the second peak in early August suggest that *D. acuminata* kept aggregated in the top water layer. This suggestion agrees with observations in recent years on the vertical distribution of this species during cell cycle studies and a couple of 2-week spring cruises in the Galician Rías and adjacent shelf [42,64]. In contrast, the even vertical distribution of toxin content when the bloom was dominated by *D. acuta* (from mid-August to November), suggests a daily vertical migration of the species within the depth range of the mussel ropes (2–12 m).

Recently, Díaz et al. [33] proposed a conceptual model based on a 29-year (1985–2013) time series of weekly observations, to explain the seasonal variability of *D. acuminata* and *D. acuta* in the Galician Rías Baixas. According to this model, years with exceptional summer blooms of *D. acuta* (such as 1989 and 1990), or even worse, with very intense autumn blooms, following spring-summer blooms of *D. acuminata*, have more severe socioeconomic impacts. This is explained by the extended duration of the *Dinophysis* bloom season, which causes a longer period of harvesting bans. The latter scenario is worsened when the autumn blooms of *D. acuta* end very late in the year, when phytoplankton is scarce and mussels take much longer time to eliminate the toxins. That was the case in 2005, when lipophilic toxins accumulated until mid-November did not clear until March 2006 [30]. In addition, toxin analyses at the monitoring center are more complex when mussels are exposed to *D. acuta* than to *D. acuminata*, and DTX2 takes longer than OA to be eliminated.

In summary, the development of *D. acuta* blooms, following those of *D. acuminata*, may represent the worst scenario for the shellfish producers in terms of duration of harvesting bans and the complexity added to the regulation LC-MS analyses of lipophilic toxins. Vertical heterogeneities in toxin distribution stress the importance of appropriate sampling strategies including sample collection at different depth of the mussel ropes.

## 4. Conclusions

The unusual persistence of thermal stratification for 2 months, combined with moderate upwelling during the summer of 1990 and presumably the abundance of prey in the Galician-Portuguese shelf (northwest Iberia), was associated with a northwards shift in the mesoscale distribution of *D. acuta*. Cell maxima of this species, restricted to a 20 km-wide band, on the shelf, between the 50 and −130 m isobaths, and vertically segregated from the co-occurring *G. catenatum*, were observed at the depth of maximal thermal gradient. Conditions associated with the overlap of summer populations of *D. acuminata* and *D. acuta* in 1990 in the Galician Rías Baixas provided new insights into the niche-partitioning of two mixotrophs sharing the same ciliate prey and where the concentrations of okadaic acid in raft mussels can be used as an indicator of the vertical distribution of both *Dinophysis* species.

## 5. Materials and Methods 

### 5.1. Study Area

The study area, northwest Iberia (~39.5°–42.5° N; 09° W), is comprised by the Galician Rías Baixas, the northern half of the Portuguese coast and the adjacent shelf. The Galician Rías Baixas are four flooded estuaries, site of intensive raft cultivation of Mediterranean mussel (*Mytilus galloprovincialis*) with production exceeding 250 × 10^3^ t per year, and extraction of other shellfish species from natural banks [65]. Shellfish exploitation is of great socioeconomic importance in the whole region under study and chronic blooms of toxin producing microalgae cause considerable damage to the local economy [4].

### 5.2. Meteorological Data

Data on air temperature, rainfall and wind speed at Vigo airport (Peinador) were obtained from the Spanish Meteorological Agency [66]. Upwelling indexes every 6 h, from the Spanish Institute of Oceanography (IEO) [67], were estimated using model data from the US Navy’s Fleet Numerical Meteorology and Oceanography Center (FNMOC) derived from sea level pressure on a grid of approximately 1°× 1° centered at 43° N 11° W, a representative location for the study area (Figure 1B). Description of the timing, variability, intensity, and duration of coastal upwelling in the Galician Rías Baixas during 1990 was made following the model proposed by Bograd et al. [68]. In this model, the Total Upwelling Magnitude Index (TUMI) is estimated as

$$TUMI=\overset{STI}{\underset{END}{\sum }}CUI\left(t\right)$$

where CUI, the Cumulative Upwelling Index, is the sum of the daily mean upwelling index; STI, the Spring Transition Index, is the date on which CUI (integrated from January 1st) reaches its minimum value; and END is the annual maximum of CUI which marks the end of the upwelling season date (autumn transition).

### 5.3. Satellite Images

Sea surface temperature (SST) images (2 km resolution) from the Advanced Very High Resolution Radiometry (AVHRR) satellite sensor were obtained from NERC (Plymouth, UK). Although both day and night AVHRR data were available, only night time data were used, because these are not affected by reflected solar radiation and geographically varying diurnal warming [69]. It is important to note that remote sensors measure radiance emitted only over the upper optical depth, typically at a depth of ~1 m in coastal waters [70].

### 5.4. Field Sampling and Phytoplankton Analyses

Weekly sampling of phytoplankton and environmental conditions in the Rías of Vigo and Pontevedra was carried out on board R.V. *Navaz* as part of the IEO monitoring program of potentially toxic phytoplankton and environmental conditions. One pilot station on each ría was visited twice a week. Following recommendations from the International Council for the Exploration of the Sea (ICES) group of experts, water samples for phytoplankton analyses were collected since 1986 with a dividable (0–5 m, 5–10 m, and 10–15 m) hose sampler [71]. This system was recommended to sample patchy populations which may escape detection with bottle sampling at discrete depths. Samples were immediately fixed on board with acidic Lugol’s iodine solution [72]. Quantitative analyses of potentially toxic phytoplankton species were carried out according to the Utermöhl [73] method. Lugol-fixed samples were analyzed with a Zeiss Invertoscop inverted microscope (Zeiss, Jena, Germany) using the method described in Utermöhl (1931). Sedimentation columns of 25 or 50 mL were filled with water samples and left to settle for 24 h. Two transects were counted at 250 X magnification to include the smaller and more abundant species. To count larger, less abundant species (including *Dinophysis* spp.), the whole surface of the chamber was scanned at a magnification of X100, so that the detection limit was 40 and 20 cell L^−1^ when samples of 25 and 50 mL respectively were sedimented.

Weekly reports of phytoplankton distributions in 1990 and 2005 at different stations along the northern Portuguese coast (Figure 1B) were obtained from the Portuguese HAB Monitoring Programme. Additional surface water samples were collected with Nansen bottles at fixed long-term monitoring stations in Cascais (Lisbon, Portugal) and off Aveiro and preserved with neutral Lugol’s iodine solution and/or buffered formalin. Subsamples of 50–100 mL were allowed to settle for 1.5–3 d. 

Sampling on the Galician shelf in 1990 was carried out on board R.V. *Navarro* during three cruises on 13–14 and 27–28 September and 11–12 October, over shelf transects (Figure 1C–E). Water samples for phytoplankton counts, chlorophyll *a* measurements and nutrients analysis were collected with Niskin bottles. Vertical profiles of temperature and salinity where obtained with a SeaBird SBE-19 CTD. In addition, Sippican XBTs were launched along a diagonal transect from Ría de Vigo to the shelf break.

In all cases, monitoring and cruise samples fixed with Lugol’s were analyzed within a few days and a few weeks after being collected. 

### 5.5. Mussel Sampling, Processing, and HPLC Analyses of Okadaic Acid (OA)

Mussels (3–5 kg) from a fixed raft in Ría de Pontevedra were collected weekly by a scuba diver, from June 1990 to January 1991, at three depths (2, 7, and 15 m) from a mussel raft rope. Ten mussels were taken at random from each depth sample, and their digestive glands removed, weighed, and kept labeled at −20 °C until analyses.

The DSP toxins extraction was done following the Lee, et al. [74] procedure with slight modifications. For each mussel sample, 1 g of homogenized hepatopancreas was extracted with 4 mL of methanol/water 80:20. After centrifugation, 2.5 mL of the supernatant was extracted twice with 2.5 mL of hexane. One milliliter of water was added to the methanolic extract and this layer was extracted twice with 4 mL of chloroform. The final chloroform extract was made up to 10 mL, and an aliquot of 0.5 mL evaporated to dryness, and reserved for derivatization with ADAM reagent (SERVA) and OA (Boehringer) was used as standard. Characteristics of the high-performance liquid chromatography (HPLC) system were Hewlett-Packard 1050, reverse-phase Superspher 100, RP-18 (Lichro-Cart 250-4, Merck, Kenilworth, NJ, USA); mobile phase, MeCN:H_2_ O (flow 1.1 mL min^−1^); column temperature 35 °C; fluorimetric detector HP 1046 A, 365 nm excitation, and 412 nm emission wavelength. 

### 5.6. Data Analysis

CTD data analysis and representation were performed using the *oce* package [75] and maps representation using ‘maptools’ [76], both from the statistical and programming software R 2.1.12 [77] available through the CRAN repository [78]. Pathfinder SST satellite data were processed and visualized using Matlab^®^ (The MathWorks Inc., Natick, MA, USA).
